# Missed Opportunities in Implementation and Optimization of Lipid-Lowering Therapies in Very-High-Risk Patients Presenting with ST-Segment Elevation Myocardial Infarction

**DOI:** 10.3390/jcm12175685

**Published:** 2023-08-31

**Authors:** Kristen Kopp, Lukas Motloch, Alexander Berezin, Victoria Maringgele, Halyna Ostapenko, Moritz Mirna, Lukas Schmutzler, Anna Dieplinger, Uta C. Hoppe, Michael Lichtenauer

**Affiliations:** 1 Department of Internal Medicine II, Division of Cardiology, Paracelsus Medical University, 5020 Salzburg, Austria; l.motloch@salk.at (L.M.);; 2 Department of Internal Medicine II, Salzkammergut Klinikum, 4840 Vöcklabruck, Austria; 3 Internal Medicine Department, State Medical University of Zaporozhye, 69061 Zaporozhye, Ukraine; 4 Department of Psychiatry, Psychotherapy and Psychosomatics, Paracelsus Medical University, 5020 Salzburg, Austria; 5 Institute for Nursing and Practice, Paracelsus Medical University, 5020 Salzburg, Austria

**Keywords:** LDL-C, lipid-lowering therapy, ESC/EAS guidelines, STEMI, very-high risk

## Abstract

The aim of this retrospective study was to provide real-world data on lipid-lowering therapy (LLT) implementation and low-density lipoprotein cholesterol (LDL-C) target achievement in an ST-segment elevation myocardial infarction (STEMI) population, with a focus on very-high-risk patients according to European guidelines criteria. Methods: Included were all STEMI patients with available LDL-C and total cholesterol treated at a large tertiary center in Salzburg, Austria, 2018–2020 (*n* = 910), with stratification into very-high-risk cohorts. Analysis was descriptive, with variables reported as number, percentages, median, and interquartile range. Results: Among patients with prior LLT use, statin monotherapy predominated, 5.3% were using high-intensity statins, 1.2% were using combined ezetimibe therapy, and none were taking PCSK9 inhibitors at the time of STEMI. In very-high-risk secondary prevention cohorts, LLT optimization was alarmingly low: 8–22% of patients were taking high-intensity statins, just 0–6% combined with ezetimibe. Depending on the very-high-risk cohort, 27–45% of secondary prevention patients and 58–73% of primary prevention patients were not taking any LLTs, although 19–60% were actively taking/prescribed medications for hypertension and/or diabetes mellitus. Corresponding LDL-C target achievement in all very-high-risk cohorts was poor: <22% of patients had LDL-C values < 55 mg/dL at the time of STEMI. Conclusion: Severe shortcomings in LLT implementation and optimization, and LDL-C target achievement, were observed in the total STEMI population and across all very-high-risk cohorts, attributable in part to deficits in care delivery.

## 1. Introduction

Cardiovascular diseases (CVDs) claim some 17.9 million lives worldwide annually [1] and circa 1.7 million lives in the European Union alone [2]. CVDs remain the leading cause of morbidity and mortality, posing a considerable burden for both the individual patient and on healthcare systems. The European Heart Network estimated in 2021 that CVD is the highest component of healthcare costs in EU member states, consuming roughly 16% of healthcare budgets, and exceeding EUR 200 billion in total annual costs [3]. Acute coronary syndrome is one of the most severe manifestations of CVD. While causes of myocardial infarction are multifactorial, atherothrombotic coronary artery disease remains the root cause of type I myocardial infarction [4]. Fundamental to the development of atherosclerotic cardiovascular disease (ASCVD) is retention of low-density lipoprotein cholesterol (LDL-C) and other cholesterol-rich apolipoprotein B-containing lipoproteins within artery walls. A large body of evidence has causally linked increased LDL-C values to ASCVD development and, inversely, has correlated lower LDL-C values with lower risk of future adverse cardiovascular (CV) events [5,6]. The past decade has seen considerable research as well as development of new therapies for treatment of atherosclerosis, primarily through targeted reduction in LDL-C levels to reduce the risk of ASCVD [7,8,9]. Thus, the European Society of Cardiology (ESC) and the European Atherosclerosis Society (EAS) jointly issued Guidelines for the Management of Dyslipidemias in 2016, which were upgraded in 2019, setting specific primary and secondary prevention LDL-C treatment targets, implementing a risk estimation scoring system to guide strategies for patient-tailored LDL-C reduction and issuing recommendations for evidence-based, lipid-lowering therapies (LLTs) [10,11]. 

Guideline-recommended LDL-C target levels are based upon total individual CV risk with necessary follow-up evaluation of treatment response, as response varies among individuals [6]. While both Guidelines provide a scoring system (Systematic Coronary Risk Estimation, SCORE) to calculate 10-year cumulative risk of a fatal CVD event, certain patient groups are identified as very-high risk without need for risk calculation. Very-high-risk patients are always targeted for rigorous LDL-C-lowering and lifestyle intervention. 

The 2016 Guidelines recommended an LDL-C target of <70 mg/dL (<1.8 mmol/L) or ≥50% reduction in baseline LDL-C (if LDL-C between 70 and 135 mg/dL) for very-high-risk patients. In 2019, the Guidelines were upgraded with a more stringent LDL-C goal of <55 mg/dL (1.4 mmol/L) and ≥50% LDL-C reduction from baseline, thus promoting “the lower the better” strategy. Secondary goals of non-HDL-C < 100 mg/dL and <85 mg/dL are recommended for very-high-risk patients in both Guidelines, respectively [10,11]. 

ESC/EAS Guidelines describe gaps in evidence with respect to implementation of a combination of LDL-C-lowering strategies and respective attainment of LDL-C goals among very-high-risk patients in real-world practice. Thus, the aims of this study are (a) to assess guideline-recommended LLT use and (b) to determine LDL-C target achievement in a real-world STEMI population with focus on patients meeting very-high-risk criteria at presentation for STEMI.

## 2. Materials and Methods

### 2.1. Study Design

**Figure 1:**
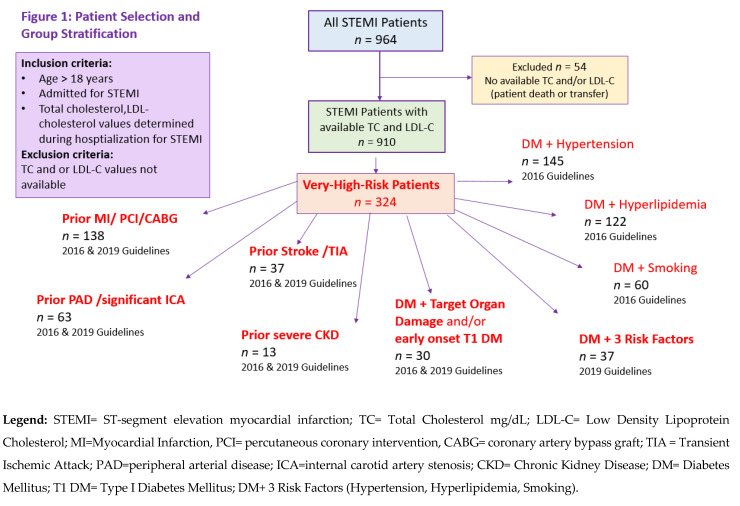
Patient selection and group stratification.

All patients (*n* = 964) presenting with STEMI between 1 January 2018 and 31 December 2020 at a single, large tertiary center in Salzburg, Austria were screened for this retrospective study. Included in the study were STEMI patients ≥ age 18, with available total cholesterol (TC, mg/dL) and LDL cholesterol (LDL-C, mg/dL) values drawn during baseline hospitalization for STEMI (*n* = 910). Excluded were patients with no available TC and/or LDL-C values (*n* = 54). Figure 1 depicts patient selection and group stratification.

Those patients with available TC and LDL-C values (*n* = 910) were screened for very-high-risk characteristics according to criteria outlined in the 2016 and 2019 *ESC/EAS* Guidelines for the Management of Dyslipidemias, the guidelines in place for the time period considered, then stratified into very-high-risk cohorts. A detailed description of very-high-risk criteria as defined in the 2016 and revised 2019 ESC/EAS Guidelines is found in Supplementary Materials Figure S1. Achievement of guideline-recommended LDL-C targets (2016, 2019) was analyzed in the total STEMI population, in the subset of STEMI patients with a very-high-risk profile (*n* = 324), and in each very-high-risk patient cohort. 

Prior lipid-lowering therapy (LLT) in use at the time of admission for STEMI was captured. High-intensity statin use was defined as current daily use of atorvastatin 40 or 80 mg, or rosuvastatin 20 or 40 mg. Moderate/low-intensity statin use was defined as current daily use of lower doses of atorvastatin <40 mg/day, rosuvastatin <20 mg/day, or any other statins/doses (simvastatin, fluvastatin, and pravastatin in our study population). Also captured was ezetimibe use in combination with statins or alone, use of PCSK9 inhibitors (PCSK9is), as well as use of any other lipid-lowering therapy (in our study only fibrate use was observed). 

### 2.2. Ethics Declaration

This study was approved by the State of Salzburg Ethics Commission (EK-Nr. 1038/2021). Data were handled according to the principles as outlined in the Declaration of Helsinki and Good Clinical Practice (ICH-GCP).

### 2.3. Data Extraction

Data were extracted from the ORBIS electronic medical records system (Agfa Healthcare, Version 08043301.04110DACHL) and the medical archiving system (Krankengeschichtsarchiv System, Uniklinikum Salzburg, Softworx by Andreas Schwab ^TM^, 2008) of the University Clinic Salzburg (Austria) using patient charts, admission, discharge, and laboratory reports from the hospitalization for STEMI. Data were entered pseudo-anonymously into an Excel database.

### 2.4. Measurement of LDL-C 

LDL-C was analyzed for all patients at the University Institute for Medical-Chemical Laboratory Diagnostics at University Clinic Salzburg. The Friedewald formula was used for calculation of plasma LDL-C concentration when triglyceride levels were <275 mg/dL; otherwise, a direct method of measurement of LDL-particle number was applied using a c702 module of the Roche Cobas^®^ 8000 analyzer (Roche Diagnostics Mannheim, Germany) according to the current manufacturer’s instructions for measurement of LDL-C. According to guidelines, both calculated and direct measurements of LDL-C show good agreement [11]. However, the reliability of the Friedewald LDL-C calculation may be influenced by a non-fasting state, and plasma LDL-C and LDL particle concentration can also become discordant in patient groups with certain conditions (i.e., diabetes, hypertriglyceridemia); therefore, analysis of non-HDL-C is also recommended and was performed [11]. 

### 2.5. Statistical Analyses

Data were summarized for the entire STEMI cohort and for each very-high-risk patient cohort. SPSS Statistical Packages for Social Sciences (SPSS) Version 27 (IBM SPSS statistics, Armonk, New York, NY, USA) was used for analysis. GraphPad Prism version 9 (GraphPad Software, San Diego, CA, USA) was used for data presentation. For categorical variables, the number and percentage of patients are given. A Shapiro–Wilk Test confirmed unequal distribution of data; thus, continuous data are reported with median and interquartile range (IQR).

## 3. Results

### 3.1. Total STEMI Population, Very-High-Risk Patient Subset

Table 1 illustrates basic patient characteristics for the entire STEMI cohort, (*n* = 910) and for the subset of very-high-risk patients (*n* = 324). The total STEMI population had a median age of 62 years (IQR 55, 72), with an older median age of 66 years (IQR 58, 75) among very-high-risk patients. Men comprised 73% and women 27% of the total STEMI population, and 74%, and 26% of the very-high-risk population, respectively. With respect to behavioral risk factors, high active smoking rates were observed in the entire STEMI population (42.1%) and in the very-high-risk subset (36.1%). Median BMI of the STEMI cohort was 26.7, and was 27.5 in the very-high-risk cohort, thus meeting the WHO classification of overweight (BMI 25 to <30 kg/m^2^). The majority of patients showed the presence of classic CV risk factors, such as prior known hypertension (67% of the total STEMI population; 81.8% in the very-high-risk cohort), hyperlipidemia (66.4%; 73.8%), and diabetes mellitus (18.9%; 51.2%). A total of 43.5% of all STEMI patients and 62% of all very high-risk patients were taking antihypertensive therapy at the time of STEMI. Among diabetes mellitus patients, 65.1% of all STEMI patients and 65.7% of very-high-risk STEMI patients were taking medication for diabetes mellitus at presentation for STEMI. 

In contrast, just 16.6% of all STEMI patients were taking LLTs at the time of STEMI. Among the very-high-risk patient subset, 36.1% were taking LLTs at the time of STEMI. With respect to statin intensity, only 5.3% of the total STEMI cohort were taking high-intensity statins, with just 1.2% taking high-intensity statins in combination with ezetimibe. Among very-high-risk patients, 13.9% were taking a high-intensity statin at the time of STEMI, only 3.1% in combination with ezetimibe. Ten percent of the total population and 19% of very-high-risk patients were taking moderate-/low-intensity statins at presentation for STEMI, just 1.2% and 0.6% taking a combination with ezetimibe, respectively. No patients were taking PCSK9 inhibitors at presentation for STEMI. Eight patients (0.9%) in the total STEMI cohort had documented statin intolerance, which therefore does not explain the observed severe LLT implementation deficits.

With respect to LDL-C targets, both guideline-recommended LDL-C cutoffs, <70 mg/dL for 2016 and <55 mg/dL for 2019, have been presented for the total STEMI and all very-high-risk population subsets due to guideline LDL-C cutoff changes that occurred during the time period under consideration. Regarding LDL-C measurement, the Friedewald equation was applied in 94.2% of the STEMI population, while in 5.8% of patients, direct LDL-C measurement was used. 

A total of 18.1% of the total population had LDL-C values < 70 mg/dL; of these 8.5% had LDL-C values < 55 mg/dL at presentation for STEMI. In the very-high-risk patient subset, 30.6% had LDL-C levels < 70 mg/dL, with 14.2% having LDL-C levels < 55 mg/dL at the time of STEMI. In patients previously treated with high-intensity statins with/without ezetimibe (48 patients; 5.3% of total cohort), the median LDL-C was 62 mg/dL (IQR 45, 76.8). Of these, 70.8% of patients on high-intensity statin treatment had achieved LDL-C values < 70 mg/dL, with just 35.4% attaining LDL-C values < 55 mg/dL. In patients previously treated with low-moderate-intensity statins with/without ezetimibe (93 patients; 10.2% of total cohort), median LDL-C was 76 mg/dL (IQR 55, 96). Here 44.1% attained LDL-C values < 70 mg/dL; of these 23.7% had LDL-C values < 55 mg/dL at the time of STEMI. In the total STEMI cohort, 17 patients (1.9%) were treated with ezetimibe in combination with statins: 11 patients taking combined ezetimibe-high-intensity statin therapy, and 6 taking a combination with moderate-/low-intensity statin therapy. Median LDL-C for patients taking combined ezetimibe–statin therapy was 63 mg/dL (IQR 40, 82). Ten patients on combined therapy (58.2%) attained LDL-C values < 70 mg/dL; of these, five patients (29.4%) achieved LDL-C values < 55 mg/dL. 

### 3.2. Very-High-Risk Patients Results by Cohort 

**Figure 2:**
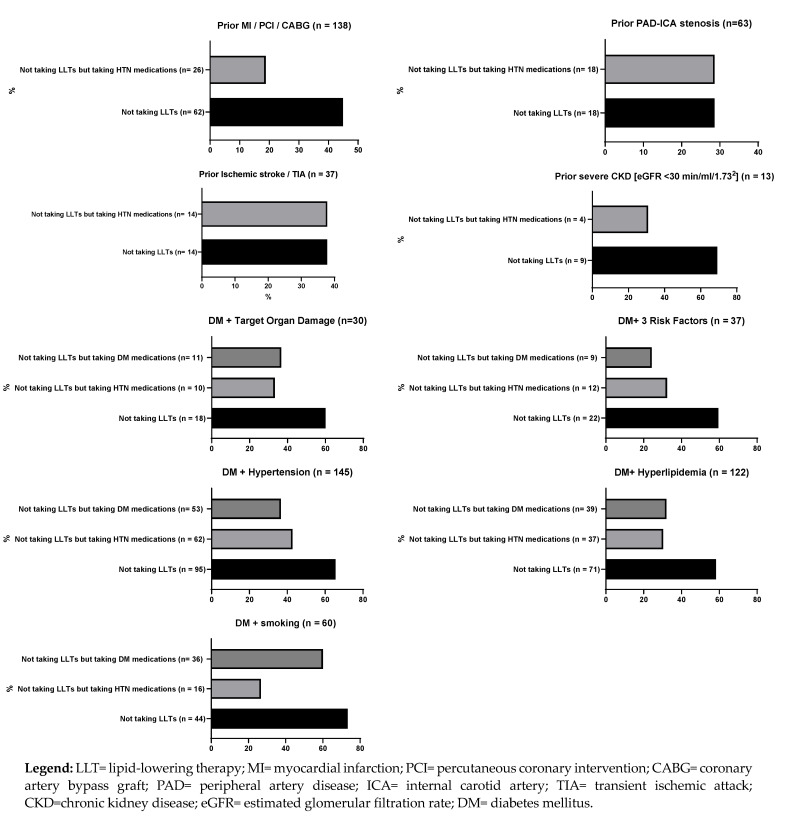
Deficits in lipid-lowering therapy implementation in very-high-risk patients.

Characteristics of each very-high-risk patient cohort are found in Table 2 and Table 3 (DM cohorts). LDL-C target achievement and LLT implementation by cohort are shown in Table 4 and Table 5 (DM cohorts). Note that both 2016 and 2019 ESC/EAS guideline-directed, risk-based cutoffs are provided for each cohort to accurately capture the guideline targets in place for the study period 2018–2020 under consideration. Deficits in LLT implementation observed in each very-high-risk cohort are presented in Figure 2, shown as percentages of very-high-risk patients not taking any LLT at the time of STEMI, as well as percentages of very-high-risk patients not taking LLTs but actively taking medications for the selected comorbidities of hypertension and/or DM, which are common in our STEMI population.

**Table 2:** Very-high-risk patient characteristics.

	Prior MI/PCI/CABG ^1^	Prior Ischemic Stroke/TIA ^2^	Prior PAD ^3^ or ICA ^4^ Stenosis	Prior Severe CKD ^5^ eGFR ^6^< 30 mL/min/1.73 ^2^
	*n* = 138	*n* = 37	*n* = 63	*n* = 13
**Age, years (median, IQR)**	65 (54.8, 75)	74 (63, 79)	73 (62, 78)	68 (62, 82)
**Men (*n*, %)/Women (*n*, %)**	110 (80), 28 (20)	23 (62), 14 (38)	39 (62 ), 24 (38)	9 (69), 4 (31)
**BMI7 kg/m^2^ (median, IQR)**	27.5 (24.7, 30.9)	26.2 (25, 28.7)	26.3 (23.8, 29.8)	25.3 (24.3, 27.1)
**Current smoker (*n*, %)**	52 (37.7)	14 (37.8)	21 (33.3)	4 (30.8)
**Medical History Diabetes (*n*, %)**	38 (27.5)	11 (29.7)	24 (38.1)	4 (30.8)
**Medical History Hypertension (*n*, %)**	114 (82.6)	30 (81.1)	53 (84.2)	10 (76.9)
**On Treatment Hypertension (*n*, %)**	92 (66.7)	25 (67.6)	42 (66.7)	9 (69.2)
**No Aspirin/P2Y12i/DOAC/VKA ^8^ (*n*, %)**	31 (22.5)	14 (37.8)	26 (41.3)	7 (53.8)
**Medical History Hyperlipidemia (*n*, %)**	117 (84.8)	23 (62.2)	45 (71.4)	8 (61.5)
**On Treatment Hyperlipidemia (*n*, %)**	76 (55.1)	14 (37.8)	28 (44.4)	4 (30.8)
**Total Cholesterol, mg/dL (median, IQR)**	153 (125, 201)	151 (127, 175)	155 (131, 200)	166 (140, 226.5)
**Triglycerides, mg/dL (median, IQR)**	112 (78, 161)	90 (67.5, 128)	110 (80, 158)	122 (94, 158)
**HDL-Cholesterol ^9^, mg/dL (median, IQR)**	46 (37, 56)	52 (40,61)	44 (36, 61)	40 (31.3, 49)
**Non-HDL-Cholesterol, mg/dL (median, IQR)**	104 (76, 150)	104 (72, 129)	109 (82.3, 152.8)	121 (78, 163)
**LDL-Cholesterol ^10^, mg/dL (median, IQR)**	**81 (59, 121)**	**86 (57, 109)**	**83 (63, 115)**	**107 (70, 157)**

Legend: ^1^ MI = Myocardial Infarction, PCI = percutaneous coronary intervention, CABG = coronary artery bypass graft; ^2^ TIA = Transient Ischemic Attack; ^3^ PAD = peripheral arterial disease; ^4^ ICA = internal carotid artery stenosis; ^5^ CKD = Chronic Kidney Disease, ^6^ eGFR = estimated Glomerular filtration rate; ^7^ BMI = Body Mass Index; ^8^ P2Y12i = P2Y12 inhibitor, DOAC = Dual Oral Anticoagulants, VKA = Vitamin K Antagonist; ^9^ HDL = High-density Lipoprotein; ^10^ LDL = Low-density lipoprotein.

**Table 3:** Very-High-Risk Diabetes Mellitus Patient Characteristics (2019 & 2016 ESC/EAS Guidelines Criteria ^1^).

	Diabetes with Target Organ Damage ^2^, or Early Onset T1DM ^3^	Diabetes + 3 Risk Factors ^4^	Diabetes + Hypertension	Diabetes + Hyperlipidemia	Diabetes + Smoking
	*n* = 30	*n* = 37	*n* = 145	*n* = 122	*n* = 60
**Age, years (median, IQR)**	74 (63, 78)	58 (53, 62.5)	68 (58, 75)	67 (59, 75)	58 (53, 64.8)
**Men (*n*, %), Women (*n*, %)**	19 (63), 11 (37)	29 (78), 8 (22)	101 (70), 44 (30)	83 (68), 49 (32)	49 (82), 11 (18)
**BMI ^5^ (median, IQR)**	27.7 (24.5, 32.2)	28.5 (25.5, 31.8)	28.5 (26, 32)	28.4 (26.3, 32.1)	28.4 (25.7, 31.4)
**Current smoker (*n*, %)**	8 (26.7)	37 (100)	44 (30.3)	45 (36.9)	60 (100)
**Medical History Hypertension (*n*, %)**	27 (90)	37 (100)	145 (100)	106 (86.9)	44 (73.3)
**On Treatment Hypertension (*n*, %)**	22 (81.5)	27 (73)	111 (76.6)	86 (70.5)	30 (68.2)
**Medical History Hyperlipidemia (*n*, %)**	20 (66.7)	37 (100)	106 (73.1)	122 (100)	46 (76.7)
**On Treatment Hyperlipidemia (*n*, %)**	12 (40)	15 (40.5)	50 (34.5%)	51 (41.8)	16 (26.7)
**On Treatment Diabetes Mellitus (*n*, %)**	23 (76.7)	20 (54.1)	92 (63.5%)	79 (64.8)	79 (64.8)
**Total Cholesterol, mg/dL (median, IQR)**	145 (128, 191)	177 (143, 209)	166 (135, 202.5)	180 (149.5, 211)	174 (143.3, 204)
**Triglycerides, mg/dL (median, IQR)**	128 (105, 204)	146 (107.5, 227.5)	138 (102.5, 205)	144 (105, 227.8)	147 (108, 216.3)
**HDL-Cholesterol ^6^, mg/dL (median, IQR)**	41 (35, 57)	40 (32, 47.5)	42 (35, 53)	43.5 (36, 54)	40.5 (32, 47.8)
**Non-HDL-Cholesterol, mg/dL (median, IQR)**	99.5 (83.8, 145.5)	142 (99, 174)	122, (90, 157)	139 (98.8, 167.8)	136.5 (99, 164.5)
**LDL-Cholesterol ^7^, mg/dL (median, IQR)**	**72.5 (58.3, 101.8)**	**104 (75.5, 145)**	**91 (64.5, 129)**	**106 (72.8, 137.3)**	**101.5 (78.3, 132)**
**HbA1C ^8^ % (median, IQR)**	6.9 (6.4, 8.3)	5.5 (5.3, 5.7)	5.5 (5.3, 5.9)	5.5 (5.2, 5.8)	5.5 (5.3, 5.7)

Legend: ^1^ ESC/EAS Guidelines = European Society of Cardiology (ESC)/European Atherosclerosis Society (EAS) Guidelines for the Management of Dyslipidemias (2016 and 2019); ^2^ Both 2016 & 2019 Guidelines define Diabetes Mellitus with Target Organ damage (2016 proteinuria; 2019 also, microalbumiuria, retinopathy); ^3^ T1DM = Type 1 Diabetes Mellitus; ^4^ DM plus 1 Risk Factor such as smoking, hypertension, dyslipidemia (2016 Guidelines), 3 Risk Factors (2019 Guidelines) not specifically defined thus 2016 risk factors used; ^5^ BMI = Body Mass Index; ^6^ HDL = High-density Lipoprotein; ^7^ LDL = Low-density lipoprotein; ^8^ HbA1C = Glycated hemoglobin (A1C).

**Table 4:** ESC/EAS ^1^ Guideline LDL-C Target Achievement and Lipid-lowering Therapy Uptake in Very-High-Risk Patients.

	Prior MI/PCI/CABG ^2^	Prior Ischemic Stroke/TIA ^3^	Prior PAD ^4^ or ICA ^5^ Stenosis	Prior severe CKD ^6^ eGFR7 < 30mL/min/1.73 ^2^
	*n* = 138	*n* = 37	*n* = 63	*n* = 13
**Achieving 2016 LDL-C Guideline Targets**				
**<70 mg/dL (*n*, %)**	59 (42.8%)	13 (35.1%)	33 (34.9%)	3 (23.1%)
**Achieving 2019 LDL-C Guideline Targets**				
**<55 mg/dL (*n*, %)**	29 (21.01%)	7 (18.9%)	7 (11.1%)	0
On High-Intensity Statin * (*n*, %)	33 (15.9%)	4 (10.8%)	5 (7.9%)	1 (7.7%)
**On High-Intensity Statin + Ezetimibe^+^ (*n*, %)**	8 (5.8%)	2 (5.4%)	0	1 (7.7%)
**On Moderate/Low-Intensity Statin ^+^ (*n*, %)**	39 (28.3%)	9 (24.3%)	22 (34.9%)	3 (23.1%)
**On Mod. /Low-Intensity Statin + Ezetimibe (*n*, %)**	4 (2.9%)	0	0	0

Legend: ^1^ ESC/EAS Guidelines = European Society of Cardiology (ESC)/European Atherosclerosis Society(EAS) Guidelines for the Management of Dyslipidemias (2016 and 2019); ^2^ MI = Myocardial Infarction, PCI = percutaneous coronary intervention, CABG = coronary artery bypass graft; ^3^ TIA = Transient Ischemic Attack; ^4^ PAD = peripheral arterial disease; ^5^ ICA = internal carotid artery stenosis; ^6^ CKD = Chronic Kidney Disease, ^6^ eGFR = estimated Glomerular filtration rate; * High Intensity statins: Atorvastatin ≥ 40 mg, Rosuvastatin ≥ 20 mg; ^+^ Moderate/low Intensity statins: atorvastatin < 20 mg, rosuvastatin < 20 mg, or all other statins/doses such as simvastatin, pravastatin, fluvastatin.

**Table 5:** ESC/EAS ^1^ Guideline LDL-C Target Achievement and Lipid-lowering Therapy Uptake in Very-High-Risk Patients with Diabetes Mellitus (2019 & 2016 ESC/EAS Guidelines Criteria).

	Diabetes with Target Organ Damage ^2^, or Early Onset Diabetes Mellitus I	Diabetes + 3 Risk Factors ^3^	Diabetes + Hypertension	Diabetes + Hyperlipidemia	Diabetes + Smoking
	*n* = 30	*n* = 37	*n* = 145	*n* = 122	*n* = 60
**Achieving 2016 LDL-C Guideline Targets**					
**<70 mg/dL (*n*, %)**	13 (43.3%)	8 (21.6%)	43 (29.7%)	28 (23%)	11 (18.3%)
**Achieving 2019 LDL-C Guideline Targets**					
**<55 mg/dL (*n*, %)**	5 (16.7%)	1 (2.7%)	21 (14.5%)	16 (13.1%)	2 (3.3%)
On High-Intensity Statin * (*n*, %)	3 (10%)	8 (21.6%)	19 (13.1%)	19 (15.6%)	8 (13.3%)
**On High-Intensity Statin + Ezetimibe (*n*, %)**	0	1 (2.7%)	2 (1.4%)	2 (1.6%)	1 (1.7%)
**On Moderate/Low-Intensity Statin ^+^ (*n*, %)**	9 (30%)	7 (18.9%)	27 (18.6%)	28 (22.95%)	6 (10%)
**On Mod. /Low-Intensity Statin + Ezetimibe (*n*, %)**	1 (3.3%)	1 (2.7%)	1 (0.7%)	2 (1.6%)	0
**On Ezetimibe Alone (*n*, %)**	0	0	2 (1.4%)	1 (0.8%)	1 (1.7%)
**On PCSK9^4^ inhibitors (*n*, %)**	0	0	0	0	0
**Documented Statin Intolerance (*n*, %)**	0	1 (2.7%)	3 (2.1%)	3 (2.5%)	1 (1.7%)

Legend: ^1^ Guidelines = European Society of Cardiology (ESC)/European Atherosclerosis Society (EAS) Guidelines for the Management of Dyslipidemias (2016 and 2019); ^2^ Both 2016 & 2019 Guidelines define Diabetes Mellitus with Target Organ (2016 proteinuria; 2019 also, microalbumiuria, retinopathy); ^3^ DM plus 1 Risk Factor such as smoking, hypertension, dyslipidemia (2016 Guidelines), 3 Risk Factors (2019 Guidelines) not specifically defined thus 2016 risk factors used; * High Intensity statins: atorvastatin > 40 mg, rosuvastatin > 20 mg; ^+^ Moderate/low Intensity statins: atorvastatin < 20 mg, rosuvastatin < 20 mg, or all other statins/doses such as simvastatin, pravastatin, fluvastatin; ^4^ PCSK9 inhibitors = proprotein convertase subtilisin/kexin type 9.

#### 3.2.1. Very-High-Risk Patients with Prior MI and/or Coronary Intervention

With respect to prior ASCVD, 138 patients (15.2% of total cohort) (110 males, 28 females) had medical history of prior myocardial infarction and/or coronary intervention, either with percutaneous coronary intervention (PCI) or coronary artery bypass grafting (CABG). These very-high-risk ASCVD patients therefore qualified for rigorous secondary prevention management of lifestyle factors and targeted LDL lowering. The median age of patients was 65 years (IQR 54.8, 75), median BMI was 27.5 (24.7, 30.9), with nearly three-quarters overweight, and 37.7% were active smokers at the time of STEMI. The median LDL cholesterol for this very-high-risk cohort was 81 mg/dL (IQR 59, 121) and median non-HDL-C was 104 mg/dL (IQR 76, 150). A total of 42.8% had LDL-C values < 70 mg/dL; of these, 21% had LDL-C values < 55 mg/dL at presentation for STEMI. 

Regarding LLT implementation, 15.9% of patients were taking high-intensity statins, with just 5.8% taking a combination with ezetimibe. A total of 28.3% of patients were taking moderate-/low-intensity statins at the time of STEMI; of these, 2.9% in combination with ezetimibe. Four patients (2.9%) had a documented statin intolerance. No patients were taking PCSK9is.

Among patients with prior MI/coronary intervention, 44.9% were not taking any LLTs at the time of STEMI. A total of 18.8% were not taking any LLTs but were, however, actively taking medications for hypertension (Figure 2). Similarly, 14.5% were taking diabetes medications, but not taking LLTs at the time of STEMI. A total of 79% of these very-high-risk patients did not meet stringent LDL-C < 55 mg/dL guideline targets. Note that 22.5% of patients with prior MI/coronary intervention were not taking prior Aspirin/P2Y12 inhibitors/Direct-acting Oral Anticoagulants DOAC/Vitamin K antagonists at the time of presentation for STEMI.

#### 3.2.2. Very-High-Risk Patients with Prior Ischemic Stroke/TIA

Thirty-seven patients (4.1%) (23 males, 14 females) had prior medical history of cerebral vascular disease, either ischemic stroke or TIA. The population was older (median age 74 years (IQR 63, 75)) and overweight, with median of BMI 26.2 (IQR 25, 26.7), and 37.8% were actively smoking at the time of STEMI. The median LDL cholesterol in this cohort was 86 mg/dL (IQR 57, 109) and median non-HDL-C was 104 mg/dL (IQR 76, 150). Just 35.1% had LDL-C values < 70 mg/dL and just 18.9% with LDL-C values < 55 mg/dL at the time of STEMI. 

Prior LLT usage was low at presentation: 10.8% were taking high-intensity statins, 5.4% in combination with ezetimibe (Table 4).

A total of 24.3% were using monotherapy moderate-/low-intensity statins, none in combination with ezetimibe. Of these very-high-risk patients, 37.8% were not taking any LLTs at the time of STEMI, although all were actively taking medications for hypertension. A total of 13.5% were taking diabetes medications but not taking LLTs at the time of STEMI. Just over 80% of prior ischemic stroke/TIA patients did not meet the strict LDL-C guideline target < 55 mg/dL at the time of STEMI.

#### 3.2.3. Very-High-Risk Patients with PAD/Significant ICA

Regarding prior vascular disease, 63 patients (6.9%) (39 males, 24 females) had medical history of either peripheral arterial disease or significant internal carotid artery stenosis confirmed by imaging. Median age was 73 years (IQR 62, 78), median BMI was 26.3 (23.8, 29.8), and 33.3% were active smokers. The median LDL-C was 88 mg/dL (IQR 63, 115) and median non-HDL-C was 109 mg/dL (IQR 82.3, 152.8). Just 7.9% of patients in this cohort were using high-intensity statins, and just 34.9% were taking moderate-/low-intensity statins at the time of STEMI. No patient in this cohort was taking a combination treatment with ezetimibe. 

Consequently, just 35% of patients in this cohort had LDL-C values < 70 mg/dL, with only 11% having LDL-C values < 55 mg/dL at presentation for STEMI. A total of 28.6% of these very-high-risk patients were not taking any LLTs at the time of STEMI, yet all were actively taking medications for hypertension. Additionally, 11% were taking anti-diabetes medications, but not taking LLTs at the time of STEMI. A total of 81% of very-high-risk PAD/significant ICA patients did not meet the strict LDL-C guideline target < 55 mg/dL at the time of STEMI.

#### 3.2.4. Very-High-Risk Patients with Severe CKD

A small group of 13 patients (1.4% of the total cohort, 9 males, 4 females) had medical history of severe chronic kidney disease (CKD) with eGFR < 30 min/mL/1.73 m^2^ without dialysis at presentation for STEMI. 

The cohort had a younger median age (68 years, IQR 62,82), median BMI was 25.3 (IQR 24.3, 27.1), and 30.8% were active smokers. The median LDL-C was 107 mg/dL (70, 157) and median non-HDL-C was 121 mg/dL (IQR 78, 163) at the time of presentation. Regarding use of LLT therapies upon admission, one patient was taking high-intensity therapy with ezetimibe, 23.1% were taking moderate-/low-intensity therapy, and none were treated with combination ezetimibe. A total of 23.1% of patients in this very-high-risk population had LDL-C values < 70 mg/dL at presentation; no patient had LDL-C values < 55 mg/dL at the time of STEMI. Yet, 69.2% were not taking any LLTs at presentation; of these, 30.8% were actively taking hypertension medications. A total of 23% were taking diabetes medications but no LLTs at presentation for STEMI.

#### 3.2.5. Very-High-Risk Patients with Diabetes Mellitus with Target Organ Damage and/or Early Onset Type I DM (2016 and 2019 Criteria)

Table 3 shows patient characteristics for very-high-risk DM cohorts and Table 5 presents LDL-C target achievement and LLT implementation in DM patients with a very-high-risk profile at the time of STEMI. Figure 2 illustrates LLT implementation deficits, also in DM very-high-risk cohorts.

Thirty patients (19 men, 11 women; 3.3% of entire cohort; 17.4% of total DM cohort) had prior target organ damage and/or early onset type 1 diabetes mellitus. Their median age was older (74, IQR 63, 78) and median BMI was 27.7 (IQR 24.6, 32.2), they were overweight with over one-quarter obese, and 26.7% were active smokers at the time of STEMI. 

Their median LDL cholesterol was 72.5 mg/dL (IQR 58.3, 101.8) and median non-HDL-C was 99.5 mg/dL (IQR 83.8, 145.5). This cohort had the highest levels of LDL-C target achievement among diabetic patients. 

Refer to Table 5 for LDL-C target achievement and LLT uptake in all DM patient cohorts. At presentation, 43.3% had LDL-C values < 70 mg/dL; of these, 16.7% had LDL-C values < 55 mg/dL. The median HbA1C value in this very-high-risk cohort was 6.9% (IQR 6.4, 8.3), uncontrolled in >25% of patients. Just 10% of these very-high-risk DM patients were taking high-intensity statins, none in combination with ezetimibe at the time of STEMI. A total of 30% were taking moderate-/low-intensity statins at the time of STEMI, with just one patient taking combined ezetimibe. 

Of these very-high-risk DM patients, 60% were not taking any LLTs at the time of STEMI. A total of 33.3% were not taking any LLTs but were actively taking medications for hypertension (Figure 2). A total of 36.7% were taking diabetes medications, but not taking LLTs at the time of STEMI. A total of 83% of this very-high-risk population did not meet stringent LDL-C guideline targets < 55 mg/dL at presentation for STEMI.

#### 3.2.6. Very-High-/High-Risk Patients with Diabetes Mellitus and One Risk Factor (2016 Criteria)

In our study, 145 patients had prior DM with the risk factor *hypertension*, 122 had DM with *hyperlipidemia*, and 60 DM patients were actively *smoking* at the time of STEMI. These patients were classified as very-high risk according to 2016 Guidelines, and as high risk according to 2019 Guidelines, all with a recommended LDL-C target of <70 mg/dL. While median age of DM patients with hypertension/hyperlipidemia was similar, DM plus active smoking patients were younger (58, IQR 53, 64). Median BMI was comparable in all three groups, with three-quarters of patients overweight/obese. A total of 30.3% of DM + hypertension and 37% of DM + hyperlipidemia patients were actively smoking at the time of STEMI. 

Median LDL cholesterol of 91 mg/dL (IQR 64.5, 129) and median non-HDL-C of 122 mg/dL (IQR 90, 157) was lowest among DM patients with hypertension. However, in DM patients with hyperlipidemia, and in DM patients with active smoking, more than three-quarters were out of target. Here, median LDL cholesterol was 106 mg/dL (IQR 72.8, 137.3) and median non-HDL-C was 139 mg/dL (IQR 98.8, 167.8), and median LDL cholesterol was 101.5 mg/dL (IQR 78.3, 132) and median non-HDL-C was 136.5 mg/dL (IQR 99, 164.5), respectively. HbA1C levels were controlled in all three groups with median HbA1C 5.5% (IQR 5.2–5.3, 5.7–5.9). Only 29.7% of DM + hypertension patients, 23% of DM + hyperlipidemia patients, and 18.3% of DM patients actively smoking had LDL-C values < 70 mg/dL at the time of STEMI. 

A total of 13–16% of DM patients with one risk factor were taking high-intensity statins at the time of STEMI, just 0.8–1.6% in combination with ezetimibe. A total of 10–23% were taking moderate/low-intensity statins at the time of STEMI, 1–2% in combination with ezetimibe. Depending upon the cohort, 2–3% of patients had documented statin intolerance. 

In very-high-/high-risk DM patients with hypertension, 65.5% were not taking any LLTs at the time of STEMI. Of these, 42.8% were not taking any LLTs but were actively taking medications for hypertension. A total of 36.6% were taking diabetes medications, but not taking LLTs at the time of STEMI. A total of 70.3% of patients in this cohort did not meet the LDL-C guideline target < 70 mg/dL at the time of STEMI

Among very-high-/high-risk DM patients with hyperlipidemia, 58.2% were not taking any LLTs at the time of STEMI. Of these, 32% were not taking any LLTs but were actively taking medications for hypertension. Similarly, 32% were taking diabetes medications, but not taking LLTs at the time of STEMI. A total of 77% of patients did not meet the LDL-C guideline target < 70 mg/dL at the time of STEMI. Of very-high-risk DM patients actively smoking, 73.3% were not taking any LLTs at the time of STEMI, yet 26.7% of these were actively taking medications for hypertension and 60% were taking diabetes medications. More than 80% of patients in these cohorts did not meet the LDL-C guideline target < 70 mg/dL at the time of STEMI. Note that 85–97% of DM patients with one ASCVD risk factor did not meet the newer, more stringent 2019 LDL-C cutoffs < 55 mg/dL.

#### 3.2.7. Very-High-Risk Patients with Diabetes Mellitus and Three Risk Factors (2019 Criteria)

The final cohort of very-high-risk patients assessed concerned DM patients with three major risk factors, fulfilling updated 2019 *Guideline* criteria for very-high-risk classification. A total of 37 patients (29 men, 8 women; 4.1% of total cohort; 21.5% of total DM cohort) had DM plus risk factors of hyperlipidemia, hypertension, and smoking. Median age was younger (58, IQR 53, 62.5). With respect to modifiable risk factors, median BMI was 28.5 (25.5, 31.8), with three-quarters of patients overweight/obese, and all actively smoking at the time of STEMI. The median LDL cholesterol in this very-high-risk cohort was 104 mg/dL (IQR 75.5, 145) and median non-HDL-C was 142 mg/dL (IQR 99, 174). HbA1C values were controlled (5.5%, IQR 5.3, 5.7). A total of 21.6% of these very-high-risk patients had LDL-C values < 70 mg/dL, and, of these, just one patient had an LDL-C < 55 mg/dL at presentation for STEMI. A total of 21.6% these very-high-risk DM patients were taking high-intensity statins, just 2.7% in combination with ezetimibe. A total of 18.9% were taking moderate/low-intensity statins at the time of STEMI, one in combination with ezetimibe. Of DM patients with three major risk factors, 59.5% were not taking any LLTs at the time of STEMI. Of these, 32.4% were not taking any LLTs but were actively taking medications for hypertension. A total of 36.6% were taking diabetes medications but were not taking LLTs. A total of 83.3% of patients in this very-high-risk population did not achieve the stringent LDL-C guideline target < 55 mg/dL at the time of STEMI.

## 4. Discussion

LDL-C is a causative risk factor in the development of ASCVD and is implicated in its clinical manifestations such as myocardial infarction and ischemic stroke. [5] A large body of evidence has shown that the degree of LDL-C reduction correlates to reduction in the relative risk of ASCVD events, as demonstrated in numerous large-scale RCTs evaluating the effect of LDL-C-reducing therapies on clinical events [7,8,12,13,14,15,16]. Every 18 mg/dL or 1 mmol/L absolute reduction in LDL-C translates into a reduction in all-cause mortality by roughly 10% and occurrence of major adverse vascular events by circa 22% [5]. Despite this evidence, the results of our study undoubtedly show that in real clinical practice, LLT implementation and optimization is by far from ideal, with recommended target levels for LDL-C achieved in only 8.5% of patients presenting with STEMI. 

Our study focused on a homogenous ASCVD patient population, i.e., those presenting with the adverse CVD event, STEMI. Prior LLT use and LDL-target achievement in our total STEMI population was low, with just 5.3% of patients taking a high-intensity statin at the time of STEMI, and just 10.2% taking moderate-low dose statins, with only 1.2% of these in combination with ezetimibe. Thus, underuse of combined statin–ezetimibe therapy was observed in our total STEMI population. None of our patients were taking PCSK9 inhibitors at the time of STEMI. PCSK9is entered the Austrian market in 2016, however, due to the high costs of therapy, reimbursement by social insurance carriers was restrictive and prescription allowed only for patients with documented statin inefficacy or intolerability after multiple testing. At the time of our study, prescription of PCSK9is was limited to four designated lipid centers in the state, representing an additional barrier to accessing these effective LDL-C-lowering therapies. Thus, a restrictive policy showed plausible residual effects on the uptake of PCSK9is in our patient population years after market introduction and updated guideline recommendations. 

Corresponding LDL-C guideline target achievement in our total STEMI population was also low: 18.1% of our high-risk STEMI patients had LDL-C values < 70 mg/dL; of these, just 8.5% had LDL-C values < 55 mg/dL at the time of presentation for STEMI. Thus, 81.9% and 91.5% of our total STEMI population were not in 2016 and 2019 Guideline LDL-C target range at the time of STEMI, respectively. 

Also observed in our study was that LDL-C target levels were not achieved even among those STEMI patients taking LLTs. A total of 29% and 65% of patients on high-intensity statin treatment did not meet LDL-C < 70 mg/dL and LDL-C < 55 mg/dL guideline targets, respectively. Among patients with combined ezetimibe therapy, just 58.2% had LDL-C values < 70 mg/dL, and 29.4% with LDL-C values < 55 mg/dL at the time of STEMI, suggesting LLT optimization deficits among those patients actually taking LLTs. Our findings align with observations from a large Australian population study, which retrospectively examined LDL-C goal achievement among all risk groups in 61,000 patients and found that only 36% of patients on statin therapy actually met therapeutic targets [12]. These findings thus emphasize the importance of LDL-C follow-up measurement and therapy optimization if LDL-C targets are unmet.

Other recently published European observational studies, such as Da Vinci (2018), Santorini (2020), and ESC-EORP Euroaspire V (2019), describe similar gaps between guideline recommendations and clinical practice for lipid management in Europe [13,14,17]. Both Da Vinci and Santorini trials included a heterogeneous population enrolled at primary, specialist lipid centers, as well as tertiary clinics, and showed low uptake of high-intensity statins across the spectrum of risk categories. In the Da Vinci study, just 22% of primary prevention patients were taking a high-intensity statin. Among secondary prevention patients, 51% of coronary disease patients, 39% of PAD patients, and 40% of cerebral vascular disease patients were taking high-intensity statins, with just 9% of patients taking combination ezetimibe therapy [13]. Corresponding LDL-C target attainment in Da Vinci was also low: only 54% of patients in Da Vinci met 2016 LDL-C cutoffs, and just 33% met 2019 cutoffs. Low rates of high-intensity statin uptake were also described in the Santorini study: 48.4% of very-high-risk patients in participating EU centers were taking high-intensity statins, with circa 26.4% taking a combination therapy with ezetimibe. A total of 75.5% of very-high-risk patients in the Santorini study did not meet strict 2019 LDL-C targets [14]. However, compared to findings in these two observational studies, our real-world STEMI patient population showed lower high-intensity statin and lower combined ezetimibe uptake. Consequently, lower percentages of patients in our study had LDL-C values within the guideline-recommended target range.

Especially interesting is an Austrian sub-study (*n* = 293) from the Da Vinci trial which describes 34% use of high-intensity statin and 46% use of moderate-intensity statin monotherapy in patients stemming from eight Austrian centers [15]. Again, as in the aforementioned studies, our real-world Austrian STEMI population showed lower LLT uptake for both statin intensities but mirrored the Da Vinci sub-study population in its use of statin monotherapy as the most common form of LLT, which the sub-study authors criticized as insufficient, especially for attainment of more stringent 2019 LDL-C goals. The Austrian Da Vinci sub-study described 58% and 38% achievement of the respective 2016 and 2019 guideline risk-based LDL-C goals, yet this poor achievement surpassed LDL-C target achievement observed in our STEMI population. A German Da Vinci sub-study of 421 primary and secondary prevention patients described greater gaps between ESC/EAS guideline recommendations and actual LDL-C goal achievement (46% attaining the 2016 goals, 28% the 2019 targets) compared to their Austrian counterparts, as well as predominant use of statin monotherapy, most commonly of moderate intensity in 49% of patients [16]. The heterogenous inclusion of registry patients may account for the discrepancy in findings among Da Vinci cohorts but, overall, the take-home message of inadequate LLT implementation and optimization, as well as low LDL-C target attainment among these registry patients remains the same. A multi-center European study of a physician survey characterizing LLT treatment patterns also showed low levels of therapy intensification, mirroring our results, yet variances in prescribing practices depending upon regions [18]. 

The Euroaspire-V trial evaluated LLT uptake and LDL-C-target achievement (2016 *Guideline* criteria) in ASCVD patients 6 months post ACS and/or post coronary intervention. Investigators observed that while 84% of very-high-risk patients were taking LLTs at 6 months post discharge, just 49.9% were taking a high-intensity statin, only 2.7% in combination with ezetimibe. At 6 months follow-up, 15.8% were not taking any LLTs. Uptake of PCSK9is was low at 0.4%. 2016 LDL-C targets <70 mg/dL were achieved in 29% of patients at 6 months post discharge, which authors concluded showed sub-optimal LLT implementation and control in patients with established ASCVD [17]. The Euroaspire-V registry results closely mirror findings in our very-high-risk STEMI subset when using the 2016 guideline LDL-C target cutoffs < 70 mg/dL (30.6% in our very-high-risk STEMI subset) and demonstrate gaps between risk-based, guideline-directed LLT recommendations and actual clinical practice, and consequently sub-optimal LDL-C guideline target achievement in this population.

### 4.1. Very-High-Risk Patients: LLT Implementation Deficits

Among our STEMI patients presenting with a very-high-risk profile, prior use of high-intensity statins was low in all cohorts (7.7–21.6%), and was also lower than uptake described among very-high-risk patient groups in the Da Vinci, Santorini, and Euroaspire-V studies. Prior use of moderate/low-intensity statins varied in our study by cohort (10–34.9%). Monotherapy with statins predominated, with 0–5.8% of patients taking combined statin–ezetimibe therapy, and none taking PCSK9 inhibitors. Thus, underutilization of guideline-recommended LLT implementation was observed in all very-high-risk patient cohorts. 

In our study, use of high-intensity statins was highest among DM patients with three risk factors (21.6%), as well as in patients with a prior MI or coronary intervention (15.9%), here suggesting heightened risk perception among these patients and their healthcare providers, which aligns with observations in the literature [19,20]. As the authors of one USA study observed, patients who visited a cardiology practice more often had intensification of LLTs compared to those managed by general practitioners and in other settings [21], which may account for slightly better uptake among our patients in the prior MI/PCI/CABG cohort with likely specialist care provision. Similarly, a large 66,158-patient 2018 Italian survey of statin utilization and lipid goal attainment in high- or very-high-risk CVD patients observed that statin use was highest (54%) among patients with recent ACS, an observation aligning with our finding of more common LLT use in this group [22]. Notably, despite guideline-recommended LLT intensification and expansion of therapy with ezetimibe or PCSK9is when LDL-C targets are unmet, more than three-quarters of patients in these two cohorts in our study were not taking high-intensity statins at the time of STEMI and less than 3% were taking them in combination with ezetimibe. Although prior use of moderate/low-intensity statins was more common, usage remained <30% in these cohorts, few (3%) with added ezetimibe. These findings align, yet are even lower, than the those from the Italian real-world, general practice lipids survey (26% use of low-moderate intensity statin, 7.5% combined with ezetimibe among recent ACS patients) [22]. These findings indicate that the low percentage of the patients treated with adequate doses of lipid-lowering drugs may be resulting from either poor adherence to the LLT after initial prescription, or a refusal to up-titrate the doses as well as possible healthcare deficits in patient follow-up.

Experience of a prior stroke/TIA also did not translate into adequate LLT usage. At the time of STEMI, only 11% were taking high-intensity statins, 5% with combination ezetimibe therapy, despite the class 1A guideline recommendation for rigorous lipid-lowering therapy to prevent further ASCVD events prevalent in this population. Suboptimal uptake of LLT was also observed in secondary prevention patients with diagnosed PAD or significant internal carotid artery stenosis, with just 8% taking high-intensity statins, 35% taking moderate-/low-intensity statins, and none taking expanded therapies with ezetimibe or PCSK9is at the time of STEMI. These findings again expose secondary prevention deficits in the follow-up of very-high-risk ASCVD patients. LLT implementation in stroke and PAD patients described in the Italian survey aligned with our findings, although low-/moderate-statin therapy was more common (49.4%) than the use of high-intensity statin therapy (5.5%) in their stroke patients, with nearly identical findings among PAD patients in both studies; findings which suggest differences in regional prescribing practices. 

Among very-high-risk primary prevention patients in our study, LLT use was comparably poor: in diabetic patients with one risk factor, under 20% were taking high-intensity statins, under 25% were taking moderate-/low-intensity statins, and few (0–2%) were taking combination therapy with ezetimibe at the time of STEMI. Notably, DM patients with the active risk factor of smoking showed the poorest uptake of LLTs among the primary prevention cohorts, a finding aligning with observations in the literature describing suboptimal LLT uptake among smokers [19].

The lowest statin use, for both intensities, occurred among the smallest very-high-risk cohorts, i.e., patients with prior severe CKD and DM patients with target organ damage (8 and 10%, respectively, among high-intensity statin use). However, these findings must be viewed with caution, on one hand due to the small sample size, and on the other because guidelines recommend careful up-titration of statins in patients with reduced renal function, which may have affected therapy intensification. Nonetheless, it must be reiterated that worsening renal function is associated with increased CVD risk and that use of combined statin/ezetimibe is considered a 1A guideline recommendation [10,11].

### 4.2. Very-High-Risk Patients Not Taking Any LLTs

Notably, many very-high-risk patients in our study were not taking any LLTs at all at the time of STEMI: 27–45% secondary prevention patients and 58–73% (i.e., the majority) of primary prevention patients, with the highest deficits observed in DM patients actively smoking. These findings highlight problems with risk awareness among primary prevention patients and underestimation of very-high risk by healthcare providers. Additionally, the findings expose deficits in follow-up care delivery among secondary prevention patients, albeit to a lesser extent than in patients without established ASCVD. 

In a German study of patient and physician perception of hypercholesterolemia, one-third of physicians estimated that >60% of their primary prevention patients were not receiving lipid-lowering therapy 20]. Even more severe were findings from the Ephesus trial. This multicenter, observational study performed among 1868 patients in Turkey evaluated patient understanding and perception of high cholesterol, as well as physician knowledge and awareness of lipid management strategies. While 68% of the secondary prevention patients in Ephesus had been prescribed statins (32% without therapy), just over 30% of primary prevention patients were prescribed a statin, meaning roughly 70% were without statin therapy [23]. Observations from the Ephesus trail align with sub-optimal LLT implementation described in our real-world STEMI population results. In the Ephesus study, patient perceptions and knowledge about statin treatment with respect to risk were also captured, exposing severe misconceptions, especially among primary prevention patients, in which 41% thought that statin treatment could be discontinued once LDL-C levels had normalized, versus 32% of secondary prevention patients with this understanding. Also notable in this study was lower adherence to medications in general, as well as higher statin discontinuation rates (40%) among primary prevention patients, attributed primarily to education levels and/or negative press about statins [23].

### 4.3. Very-High-Risk Patients: Low LDL-C Target Attainment

Corresponding 2016 and 2019 guideline-recommended LDL-C target achievement rates across all very-high-risk patient cohorts at the time of STEMI were under 43% and under 22%. Among STEMI patients with prior known ASCVD with prior myocardial infarction/prior PCI/CABG, or stroke/TIA, or significant PAD/ICA, attainment of the LDL-C < 70 mg/dL target was 42.8%, 35.1%, and 34.9%, and even lower for the LDL-C < 55 mg/dL target, in 21%, 18.9%, and 11.1% of patients, respectively. Target attainment among patients with severe CKD corresponded to low LLT uptake, with 23% of patients attaining the LDL-C < 70 mg/dL target and none achieving the LDL-C < 55 mg/dL LDL-C target. Among diabetic patients, attainment of the LDL-C < 70 mg/dL was observed in 43% of DM patients with target organ damage/early onset DM, but in only 18–30% of DM patients with other risk factors, with smokers notably showing the lowest LDL-C target achievement.

### 4.4. Healthcare Delivery Deficits

Several healthcare delivery deficits were observed among very-high-risk STEMI patients in our study. First, among very-high-risk patients taking LLTs at the time of STEMI, therapy had not been optimized in the majority of patients. Most were taking moderate-/low-intensity statins and were only taking statin monotherapy, despite low LDL-C target achievement. Few very-high-risk patients (0–3%) were taking combined ezetimibe therapy and none had been receiving more potent PCSK9is at the time of STEMI. As patients in Austria must physically go to their general practitioners or internists to obtain LLT prescriptions, an opportunity to measure LDL-C, determine efficacy of current LLT, and, where necessary, intensify treatment either through up-titration to maximally tolerated doses of statins and/or therapy expansion with ezetimibe or PCSK9 inhibitors has been missed.

Several studies suggest poor patient adherence and/or diminishing uptake of LLTs over time, often in conjunction with LLT side-effects, such as statin-related muscle pain [24]. However, the percentage of patients with documented statin intolerance in our study was low (1%); thus, the lack of therapy intensification and poor LLT uptake cannot be attributed solely to statin intolerance/side-effects. Notably, when current guideline-recommended therapies are not tolerated, or are not proven effective, emerging alternative classes of drugs shown to lower LDL-C, such as the small interfering RNA injectable, inclisirian, as an alternative to PCSK9is, or the ATP citrate lyase inhibitor, bempedoic acid, offer benefit for statin-intolerant patients [11].

The most important observation in our study concerns the 27–45% share of very-high-risk secondary prevention patients not taking any LLTs at the time of STEMI. Of these, however, 18.8–37.8% were actively taking and actively prescribed medications for the common comorbidity hypertension. A similar observation applies to the 58–73% very-high-risk DM patients not taking any LLTs at the time of STEMI, yet 24–60% of these were actively taking/actively prescribed medications for DM. These findings clearly implicate health care providers in deficits in LLT implementation and prescription. Either patients are not recognized as very-high risk and are not prescribed guideline-directed LLTs, prescription is simply overlooked, or potential adherence problems/potential LLT treatment side-effects are not being addressed at the time of prescription of medications for other comorbidities. Notably, secondary prevention patients have been seen by multiple healthcare providers at the time of a prior event and/or diagnosis and when obtaining prescriptions; however, LLT use in many of these very-high-risk patients was still lacking, suggesting suboptimal therapy coordination and follow-up between healthcare providers.

When considering that hypertension was the most common comorbidity and that 67% of STEMI patients and 82% of very-high-risk STEMI patients in our study were taking antihypertensive medications, coupling risk assessment, LDL-C control, and LLT prescription at the time of prescription of antihypertensive agents offers the potential to improve LLT uptake and better control ASCVD risk in these very-high-risk patients.

Identification and follow-up of very-high-risk patients is essential not just for control of LDL-C, but an opportunity for management of other ASCVD risk factors. In our study, more than three-quarters of STEMI patients were overweight/obese. The percentage of very-high-risk patients actively smoking at the time of STEMI was high, at 27–38%, exceeding both the 2019 Austrian national average of 21% and the EU average of 18.4% daily active smokers [25].

### 4.5. Limitations 

The main limitation of our study is the retrospective, single-center design, which means that our results may not reflect LLT implementation or LDL-C target achievement in STEMI populations in other EU or world regions. However, our study serves as a local quality indicator and shows severe deficits in the implementation and uptake of LLTs and poor LDL-C target achievement among very-high-risk patients, linked partly to healthcare delivery deficits, a finding which may be applicable in other regions.

Our study had some other limitations, such as lack of lipoprotein-B measurement, which is not routinely measured in STEMI patients at our hospital. Patients with a calculated SCORE ≥ 10% are considered very-high risk for 10-year fatal CVD events. However, we did not use the SCORE calculator to solely classify patients as very-high risk in our retrospective study, as we could not confirm if blood pressure measurements required for SCORE were performed in a harmonized way at presentation for STEMI. Thus, the actual number of very-high-risk patients may be underestimated. Familial hypocholesteremia (FH) was not captured as a variable in our study. While those with confirmed ASCVD were included by default, those with FH and only one major risk factor may have been missed, again possibly resulting in an underestimation of the total number of very-high-risk patients. 

A retrospective study cannot confirm a causative effect of LDL-C in excess of guideline-recommended target levels with the subsequent presentation for STEMI. However, as is well documented in the literature, LDL-C is implicated in the development of ASCVD and absolute LDL-C reductions correlate with reductions in all-cause mortality and occurrence of major adverse CV events, such as STEMI. Our study therefore only seeks to provide insights regarding LLT implementation and current lipid profiles in a real-world ASCVD patient population at the time of STEMI.

## 5. Conclusions

Our findings demonstrate severe shortcomings in routine clinical practice in terms of adequate LLT implementation and optimization with resultant LDL-C levels outside of guideline-recommended targets in the majority of patients presenting with a very-high-risk profile at the time of STEMI. Especially critical was poor LLT uptake and LLT optimization observed in very-high-risk secondary prevention ASCVD patients. Furthermore, depending on the very-high-risk cohort, up to 45% of secondary prevention patients and up to 73% of primary prevention patients were not taking any LLTs at all at the time of STEMI, although up to 42% were actively taking medications for hypertension. A similar observation applies in up to 60% of very-high-risk/high risk DM patients actively taking DM medications but not taking LLTs at the time of STEMI. Among all very-high-risk patient cohorts, these findings demonstrate suboptimal follow-up care delivery, and missed opportunities for LDL-C control, prescription and optimization of LLTs, risk discussion, and adherence support.

## Figures and Tables

**Table 1:** Patient characteristics.

	Entire STEMI Population	Very-High-Risk Patients
	*n* = 910	*n* = 324
**Age, years (median, IQR)**	62 (55, 72)	66 (58, 75)
**Men (*n*, %), Women (*n*, %)**	664 (73%), 246 (27%)	239 (74%), 85 (26%)
**BMI ^1^ kg/m ^2^ (median, IQR)**	26.7 (24.5, 29.7)	27.5 (24.7, 30.9)
**Current smoker (*n*, %)**	383 (42.1%)	117 (36.1%)
**Medical History prior Myocardial Infarction/PCI ^2^/CABG ^3^ (*n*, %)**	138 (15.2%)	138 (42.6%)
**Medical History prior Ischemic Stroke/TIA ^4^ (*n*, %)**	37 (4.1%)	37 (11.4%)
**Medical History significant PAD ^5^/ICA ^6^ stenosis (*n*, %)**	63 (6.9%)	63 (19.1%)
**Medical History Renal Insufficiency eGFR ^7^ < 30 mL/min/1.732 (*n*, %)**	13 (1.43%)	13 (4%)
**Medical History Hypertension (*n*, %)**	609 (67%)	265 (81.8%)
**On Treatment Hypertension (*n*, %)**	396 (43.5%)	203 (62.7%)
**Medical History Hyperlipidemia (*n*, %)**	604 (66.4%)	239 (73.8%)
**On Treatment Hyperlipidemia *(n*, %)**	151 (16.6%)	117 (36.1%)
**Medical History Diabetes Mellitus (*n*, %)**	172 (18.9%)	166 (51.2%)
**On Treatment Diabetes Mellitus *(n*, %)**	111 (12.2%)	109 (65.7%)
**Total Cholesterol, mg/dL (median, IQR)**	181 (152, 212)	166 (136, 205)
**Triglycerides, mg/dL (median, IQR)**	111 (81.8,160)	125 (87, 181)
**HDL-Cholesterol ^8^, mg/dL (median, IQR)**	48 (39,58)	45 (36, 56)
**Non-HDL-Cholesterol, mg/dL (median, IQR)**	130 (100, 162)	120 (85, 158)
**LDL Cholesterol ^9^, mg/dL (median, IQR)**	**107 (81, 137)**	**93 (64, 128)**
**HbA1C ^10^ % (median, IQR)**	5.6 (5.3, 5.9)	6.1 (5.5, 7.1)
On High-Intensity Statin * (*n*, %)	48 (5.3%)	44 (13.9%)
**On High-Intensity Statin + Ezetimibe ^+^ (*n*, %)**	11 (1.2%)	10 (3.1%)
**On Moderate/Low-Intensity Statin ^+^ (*n*, %)**	93 (10.2%)	62 (19.1%)
**On Mod./Low-Intensity Statin + Ezetimibe (*n*, %)**	4 (1.2%)	2 (0.6%)
**On Ezetimibe Alone (*n*, %)**	2 (0.2%)	4 (1.2%)
**On PCSK9 ^11^ inhibitors (*n*, %)**	0	0
**Documented Statin Intolerance (*n*, %)**	8 (0.9%)	4 (1.2%)
**Achieving 2016 LDL-C Guideline Targets < 70 mg/dL (*n*, %)**	165 (18.1%)	99 (30.6%)
**Achieving 2019 LDL-C Guideline Targets < 55 mg/dL (*n*, %)**	77 (8.5%)	46 (14.2%)

Legend: ^1^ BMI = Body Mass Index, ^2^ PCI = percutaneous coronary intervention; ^3^ CABG = coronary artery bypass graft; ^4^ TIA = Transient Ischemic Attack; ^5^ PAD = peripheral arterial disease, ^6^ ICA = internal carotid artery stenosis, ^7^ GFR = estimated Glomerular filtration rate; ^8^ HDL = High-density Lipoprotein; ^9^ Low-density lipoprotein; ^10^ HbA1C = glycated hemoglobin A1C; ^11^ PCSK9 inhibitors = Proprotein convertase subtilisin/kexin type 9 inhibitor. * High Intensity statins: Atorvastatin ≥ 40 mg, Rosuvastatin ≥ 20 mg; ^+^ Moderate/low Intensity statins: Atorvastatin < 20 mg, Rosuvastatin < 20 mg, or all other statins/doses, in our study: Simvastatin, Pravastatin, Fluvastatin.

## Data Availability

Blinded datasets are available upon request from the corresponding author.

## Supplementary Materials

The following supporting information can be downloaded at: https://www.mdpi.com/article/10.3390/jcm12175685/s1, Text S1: 2016 and 2019 ESC/EAS Guidelines for the Management of Dyslipidemias, Very-High-Risk Criteria.
