# Supplementary material for: Missed Opportunities in Implementation and Optimization of Lipid-Lowering Therapies in Very-High-Risk Patients Presenting with ST-Segment Elevation Myocardial Infarction
Source: J Clin Med. 2023 Aug 31;12(17):5685. doi: 10.3390/jcm12175685 (PMC10488884; doi:10.3390/jcm12175685)
Supplement: Supplementary file 1 [file jcm-12-05685-s001.zip › jcm-2544086-supplementary.pdf]

Supplement Text S1: 2016 and 2019 ESC/EAS Guidelines for the Management of Dyslipidemia Very-High Risk Criteria used in our STEMI study

Patients were stratified into cohorts according to very-high risk criteria defined in the 2016 and revised 2019 *ESC/EAS Guidelines for the Management of Dyslipidemias* [1, 2], the two guidelines in place for the time-period under consideration. Very-high risk patients were classified as those with overt, documented ASCVD, either clinical or unequivocal on imaging. Included were patients with previous myocardial infarction (MI) and/or coronary revascularization with percutaneous coronary intervention (PCI) or coronary artery bypass grafting (CABG) (n=137). Also classified as very-high risk were patients with previous stroke or transient ischemic attack (TIA) (n= 37), and those with peripheral arterial disease (PAD) or significant internal carotid artery stenosis as seen on imaging (n= 63). Patients with prior severe chronic kidney disease (CKD) and with an estimated glomerular filtration rate (eGFR) < 30 min/mL/1.73m^2 were classified as very- high risk (n=13).

Diabetes mellitus (DM) patients with evidence of target organ damage, defined as microalbuminuria, retinopathy, or neuropathy, and/or early onset Type I DM (> 20 years) were classified in both 2016 and 2019 *Guidelines* as very- high risk patients, thus previously diagnosed DM patients meeting these criteria were assessed in our study (n=30). Yet risk classification for other very-high risk DM patient groups differed by years. The 2016 Guidelines describe DM patients with just one additional risk factor as very-high risk for 10-year fatal CVD, thus diabetes mellitus patients with hyperlipidemia (n = 122), hypertension (n=145), or smoking (n=60) were included as our study evaluated all STEMI patients 2018-2020, a time period overlapping both guidelines. Following 2019 *Guideline* revision, DM plus one risk factor was down-graded to high risk only, retaining the less-stringent LDL-C target of < 70 mg/dL, applicable to each of these three DM patient cohorts.

In the 2019 *Guideline* upgrade, DM plus three major risk factors became the new requirement for very-high risk classification along with the stricter LDL-C target of < 55 mg/dL. Here 37 patients meeting the criteria DM plus 3 risk factors (hyperlipidemia, hypertension, smoking) were included in our study (n=37).

1. Catapano AL, Graham I, De Backer G *et al.* 2016 ESC/EAS Guidelines for the Management of Dyslipidaemias. *Eur Heart J* 2016;**37**:2999–3058.

2. Mach F, Baigent C, Catapano AL *et al.* 2019 ESC/EAS Guidelines for the management of dyslipidaemias: lipid modification to reduce cardiovascular risk. *European Heart Journal* 2020;**41**:111–88.
